# Incidence of SARS-CoV-2 Infection Among European Healthcare Workers and Effectiveness of the First Booster COVID-19 Vaccine, VEBIS HCW Observational Cohort Study, May 2021–May 2023

**DOI:** 10.3390/vaccines12111295

**Published:** 2024-11-19

**Authors:** Camelia Savulescu, Albert Prats-Uribe, Kim Brolin, Zvjezdana Lovrić Makarić, Anneli Uusküla, Georgios Panagiotakopoulos, Colm Bergin, Catherine Fleming, Antonella Agodi, Paolo Bonfanti, Rita Murri, Viesturs Zvirbulis, Dace Zavadska, Konstanty Szuldrzynski, Ausenda Machado, Corneliu Petru Popescu, Mihai Craiu, Maria Cisneros, Miriam Latorre-Millán, Goranka Petrović, Liis Lohur, Kyriaki Tryfinopoulou, Jonathan McGrath, Lauren Ferguson, Martina Barchitta, Anna Spolti, Katleen de Gaetano Donati, Ilze Abolina, Dagne Gravele, Vânia Gaio, Simin Aysel Florescu, Mihaela Lazar, Pilar Subirats, Laura Clusa Cuesta, Gordan Sarajlić, Marina Amerali, Jacklyn Sui, Claire Kenny, Venerando Rapisarda, Marianna Rossi, Silvia Lamonica, Dainis Krievins, Elza Anna Barzdina, Ana Palmira Amaral, Alma Gabriela Kosa, Victor Daniel Miron, Carmen Muñoz-Almagro, Ana María Milagro, Sabrina Bacci, Piotr Kramarz, Anthony Nardone, the VEBIS HCW VE Study Group

**Affiliations:** 1 Epiconcept, 75011 Paris, France; 2 European Centre for Disease Prevention and Control, 169 73 Solna, Sweden; 3 Croatian Institute of Public Health, 10000 Zagreb, Croatia; 4 Institute of Family Medicine and Public Health, University of Tartu, 50090 Tartu, Estonia; 5 National Public Health Organization (EODY), EL-15123 Athens, Greece; 6 Department of Genitourinary Medicine and Infectious Diseases (GUIDe), St. James’s Hospital, D08 NHY1 Dublin, Ireland; 7 Department of Clinical Medicine, Trinity College, D02 PN40 Dublin, Ireland; 8 Department of Infectious Diseases, University Hospital Galway, H91 YR71 Galway, Ireland; 9 Department of Medicine, University of Galway, H91 TK33 Dublin, Ireland; 10 Department of Medical and Surgical Sciences and Advanced Technologies “GF Ingrassia”, University of Catania, 95124 Catania, Italymartina.barchitta@unict.it (M.B.); 11 Azienda Ospedaliero-Universitaria Policlinico AOUP “G. Rodolico-San Marco”, 95124 Catania, Italy; venerando.rapisarda@unict.it; 12 Infectious Diseases Unit, Fondazione IRCCS San Gerardo dei Tintori, Monza—University of Milano, Bicocca, 20126 Milano, Italy; 13 Fondazione Policlinico Universitario A. Gemelli IRCCS, 00168 Rome, Italysilvia.lamonica@policlinicogemelli.it (S.L.); 14 Dipartimento di Sicurezza e Bioetica, Università Cattolica del Sacro Cuore, 00168 Rome, Italy; 15 Pauls Stradins Clinical University Hospital, 1002 Riga, Latvia; viesturs.zvirbulis@stradini.lv (V.Z.);; 16 Children Clinical University Hospital, 1004 Riga, Latvia; 17 National Institute of Medicine of the Ministry of Interior and Administration, 02-106 Warsaw, Poland; 18 Department of Epidemiology, National Institute of Health Doutor Ricardo Jorge, 1649-016 Lisbon, Portugal; 19 Victor Babes Clinical Hospital of Infectious and Tropical Diseases, 030303 Bucharest, Romania; 20 Faculty of Medicine, Carol Davila University of Medicine and Pharmacy, 020021 Bucharest, Romania; 21 National Institute for Mother and Child Care Alessandrescu Rusescu, 20382 Bucharest, Romania; 22 Institut de Recerca Sant Joan de Deu, Hospital Sant Joan de Deu, 08950 Barcelona, Spain; 23 Medicine Department, Universitat Internacional de Catalunya, 08195 Barcelona, Spain; 24 Research Group on Difficult to Diagnose and Treat Infections, IIS Aragon, Miguel Servet University Hospital, 50009 Zaragoza, Spain; mlatorre@iisaragon.es (M.L.-M.); lauraclusa@gmail.com (L.C.C.); amilagro@salud.aragon.es (A.M.M.); 25 Outpatient Department, Viljandi Hospital, 71024 Viljandi, Estonia; liis.lohur@vmh.ee; 26 Cantacuzino National Military-Medical Institute for Research and Development, 050096 Bucharest, Romania; 27 Department of Occupational Risk Prevention, Hospital Sant Joan de Deu, 08950 Barcelona, Spain; 28 Department of Clinical and Experimental Medicine, Occupational Medicine, University of Catania, 95124 Catania, Italy; 29 Centro Hospitalar Tondela-Viseu EPE, 3504-509 Viseu, Portugal; 30 Ciber of Epidemiology and Public Health CIBERESP, 28029 Madrid, Spain

**Keywords:** COVID-19, SARS-CoV-2, vaccine effectiveness, healthcare workers, Europe

## Abstract

**Background:** European countries have included healthcare workers (HCWs) among priority groups for COVID-19 vaccination. We established a multi-country hospital network to measure the SARS-CoV-2 incidence and effectiveness of COVID-19 vaccines among HCWs against laboratory-confirmed SARS-CoV-2 infection. **Methods:** HCWs from 19 hospitals in 10 countries participated in a dynamic prospective cohort study, providing samples for SARS-CoV-2 testing at enrolment and during weekly/fortnightly follow-up. We measured the incidence during pre-Delta (2 May–6 September 2021), Delta (7 September–14 December 2021), and Omicron (15 December 2021–2 May 2023) waves. Using Cox regression, we measured the relative vaccine effectiveness (rVE) of the first COVID-19 booster dose versus primary course alone during Delta and Omicron waves. **Results:** We included a total of 3015 HCWs. Participants were mostly female (2306; 79%), with a clinical role (2047; 68%), and had a median age of 44 years. The overall incidence of SARS-CoV-2 infection was 3.01/10,000 person-days during pre-Delta, 4.21/10,000 during Delta, and 23.20/10,000 during Omicron waves. rVE was 59% (95% CI: −25; 86) during Delta and 22% (1; 39) during Omicron waves. rVE was 51% (30; 65) 7–90 days after the first booster dose during the Omicron wave. **Conclusions:** The incidence of SARS-CoV-2 infection among HCWs was higher during the Omicron circulation period. The first COVID-19 vaccine booster provided additional protection against SARS-CoV-2 infection compared to primary course vaccination when recently vaccinated <90 days. This multi-country HCW cohort study addressing infection as the main outcome is crucial for informing public health interventions for HCWs.

## 1. Introduction

Healthcare workers (HCWs) are one of the eight key occupational groups considered essential for societies to function, representing 20% of the critical workforce in high-income countries [1]. Respiratory infections such as coronavirus disease (COVID-19) are the most common occupational hazards in HCWs, and can also be transmitted to vulnerable patients at risk of developing severe disease [2,3,4]. Protecting HCWs through early detection, non-pharmaceutical interventions, and vaccination leads to a reduction in transmission and prevents nosocomial infections in hospitals [5].

During the COVID-19 pandemic, by May 2021, WHO estimated 115,500 (80,000 to 180,000) deaths globally attributable to COVID-19 among HCWs, a mortality that was considered to be greatly underestimated [6]. By WHO region, the highest mortality in HCWs was reported in Europe in the first year of the pandemic [7]. COVID-19 morbidity among HCWs was also reportedly higher than in the general population [4,8]. COVID-19 vaccination in HCWs was considered one of the main pillars of pandemic control with evolving recommendations over time [9]. In the European Union and European Economic Area (EU/EEA), five spike-based vaccines were initially granted conditional marketing authorisation and were included in EU/EEA vaccination programmes with a one- or two-dose schedule (according to the vaccine), by May 2021. The first booster dose was included in the vaccination programmes beginning September 2021 [3] and the second booster dose starting in September 2022 [10].

High COVID-19 vaccine uptake was recorded in HCWs in the EU/EEA for the primary course (first and second doses), reaching a median of 86% and 80%, respectively [11], and it remained high for the first booster dose, that was mainly recommended during the SARS-CoV-2 Delta wave. Learning from the evaluation of primary course and first booster COVID-19 vaccine effectiveness in this high-risk group is crucial to shape the vaccination policies among essential workers as well as in the general population for future pandemics.

Following the deployment of COVID-19 vaccination, the European Centre for Disease Prevention and Control (ECDC) initiated the Vaccine Effectiveness, Burden and Impact Studies (VEBIS), aimed at measuring COVID-19 and influenza vaccine effectiveness (VE) in different settings including HCW [12]. The protocol used for this HCW cohort study [13] adapted the WHO protocol, using as main outcome the SARS-CoV-2 infection rather than symptomatic disease [14,15].

The rationale for the current study is the limited knowledge on first booster dose VE against symptomatic and asymptomatic infection and its variation with time. Here, we describe the HCW cohort and the incidence of SARS-CoV-2 infection, and present the results of the prospective study aimed at measuring the relative VE (rVE) of the first COVID-19 booster dose against laboratory-confirmed SARS-CoV-2 infection during the Delta and Omicron predominant circulation periods (or “waves”), using HCWs with primary course vaccination as a reference group.

## 2. Materials and Methods

We established a dynamic, prospective, longitudinal multicentre cohort among HCWs eligible for COVID-19 vaccination from 19 hospitals in 10 countries participating in the VEBIS project, following the same protocol [13]. Hospitals were invited according to their expression of interest in the study and the feasibility of regular testing using reverse-transcription Polymerase Chain Reaction (RT-PCR).

All categories of hospital HCWs (clinical, allied professional, ancillary, etc.) were eligible for recruitment. We enrolled into the cohort HCWs in whom vaccination was not contraindicated and who provided informed consent for participation. At enrolment, participating HCWs provided a nasopharyngeal or saliva sample for RT-PCR testing. They also completed an enrolment questionnaire that included demographic, clinical (vaccination history and previous infection with SARS-CoV-2), and occupation- and community-related behavioural questions. Participating HCWs also provided blood samples for serology testing.

The study participants were regularly followed up with the collection of either weekly or fortnightly respiratory samples for RT-PCR testing and a weekly questionnaire to record changes in vaccination status and potential professional and community exposures. Saliva samples were acceptable if a weekly collection schedule was followed. The study required a minimum follow-up time of 3 consecutive months for each participant. Regular contact with participants was provided by study teams in the participating hospitals to minimise the loss of follow-up.

The primary outcome was the first confirmed SARS-CoV-2 infection detected by RT-PCR in any participant during the follow-up, regardless of symptoms. Secondary outcomes were defined according to severity: asymptomatic, symptomatic, and severe COVID-19. Asymptomatic infection was defined as any participant with RT-PCR-confirmed SARS-CoV-2 infection reporting no symptoms. Symptomatic COVID-19 was defined as any participant with RT-PCR-confirmed SARS-CoV-2 infection who reported one or more of the clinical criteria conforming with the ECDC possible case definition of COVID-19 (ECDC 2021), namely, cough, fever, shortness of breath/dyspnoea, sudden onset of anosmia, and ageusia/dysgeusia, from 14 days before to 6 days after the first positive RT-PCR test. Severe COVID-19 were symptomatic cases that needed hospitalisation due to the COVID-19 episode. If a participating HCW reported testing positive for SARS-CoV-2 outside the study, the information was recovered by the study teams and included in the follow-up study questionnaire. Depending on participating site capacity, all or a proportion of SARS-CoV-2 confirmed samples with a Ct (cycle threshold) value < 30 were selected for variant identification by PCR or genetic sequencing.

We allocated HCW follow-up time to one or more of pre-Delta, Delta, or Omicron variant predominant circulation periods. As genetic sequencing data were not available at all sites, we inferred the predominant SARS-CoV-2 variant period by time, when the first cases of Delta or Omicron variant were identified in the study (when the sequencing data were available), or according to the Global Initiative on Sharing All Influenza Data (GISAID) percentage > 75% in the countries from where hospitals not sequencing the SARS-CoV-2 viruses came from. Thus, we defined the pre-Delta period as 3 May–6 September 2021, the Delta period as 7 September–14 December 2021, and the Omicron period as 15 December 2021–2 May 2023.

We performed a descriptive analysis of the cohort at participant enrolment overall and during the start of each predominant SARS-CoV-2 circulation period (pre-Delta, Delta, and Omicron) by socio-demographics (age and sex), professional roles, clinical history (smoking history, previous SARS-CoV-2 infection, underlying conditions, and vaccination status), and possible professional and personal exposures in the community (household members, use of public transport, attendance of social events, or contact with a case at home). We also described the SARS-CoV-2 cases by the same time-dependent characteristics.

We calculated the incidence of SARS-CoV-2 infection as the number of events reported during the person-time at risk. Each participant began contributing person-time at risk from the date of enrolment, 60 days after a PCR-confirmed SARS-CoV-2 infection if positive at or before enrolment, or 14 or more days after completion of a primary vaccination course, whichever was the latest. Person-time at risk ended at SARS-CoV-2 infection (the date of either the first positive RT-PCR test or first onset of symptoms, whichever was earlier), the date of end of the study for the participant, loss of follow-up, or censor date (2 May 2023).

Exposure in the rVE study was vaccination with the first booster COVID-19 vaccine dose, defined as ≥7 days after having received the first booster dose following completion of a two-dose primary course vaccine, or ≥7 days after having received the first booster dose following completion of a single-dose primary course (i.e., Jcovden, previously known as COVID-19 vaccine Janssen), in HCWs eligible to receive the first booster dose (i.e., 3 months after completion of the primary course vaccination) until they received the second booster dose. The exposure reference was primary course vaccination, defined as ≥14 days after the second dose of a two-dose vaccine, or ≥14 days after the first dose of a single-dose vaccine (i.e., Jcovden), in HCWs eligible for vaccination. Those partially vaccinated, i.e., HCWs receiving one- or two-dose vaccination until <14 days after the second dose for a two-dose schedule only, and unvaccinated HCWs were excluded from the rVE analysis. We also excluded those HCWs with a “wash-out” period of 0–13 days after the first or second dose of a vaccine and 0–6 days after the first booster. The vaccination status of HCWs was a time-changing variable. HCWs could move between the primary course vaccination and the first booster vaccination.

To estimate rVE, we compared hazard rates of SARS-CoV-2 infections among HCWs who were vaccinated with the first booster dose and those of HCWs vaccinated with primary course alone. The rVE analysis was possible for the Delta and Omicron waves only, as the first booster vaccination started to be recommended in September 2021. For this analysis, we excluded HCWs with immunocompromising conditions for whom a three-dose primary course vaccination was recommended and those with recent SARS-CoV-2 infection (<60 days). Participating HCWs that presented serologic evidence of infection during the study (anti-N testing performed in seven sites) not concordant with the virology testing were also excluded from the rVE analysis. In addition, we excluded HCWs who did not respond to enrolment or any follow-up questionnaires, and those with missing or incompatible information in age, sex, or vaccination status. We also excluded person-time if more than one virology test was missed (based on site testing frequency), and we considered an HCW lost to follow-up if more than two tests were missed. The rVE analyses were restricted to complete data.

rVE was calculated as (1-HR) × 100 where HR was the result of Cox regression, adjusted for age, sex, any underlying condition, hospital, and reported previous SARS-CoV-2 infection at enrolment. We also measured rVE by time since vaccination (7–89 or ≥90 days after the first booster dose compared to ≥90 days after primary course vaccination). Age was included in the regression model as a second-degree polynomial, based on the Akaike Information Criterion (AIC). Calendar time was used as the underlying time in the Cox regression. Statistical analysis was performed using Stata 17 (StataCorp. 2021. Stata Statistical Software: Release 17. College Station, TX, USA: StataCorp LLC.).

## 3. Results

### 3.1. Description of the Cohort

Between 2 May 2021 and 2 May 2023, a total of 3327 HCWs from 19 hospitals in 10 countries (Croatia, Estonia, Greece, Latvia, Ireland, Italy, Poland, Portugal, Romania, and Spain) participated in the multi-country VEBIS HCW study. European hospitals participated dynamically throughout the study period, according to the ethical approval timing and local requirements for active surveillance for SARS-CoV-2 infections. The median participation by month of the study was 8 hospitals (ranging between 4 hospitals in June 2021 and 12 hospitals in April 2023).

**Figure 1:**
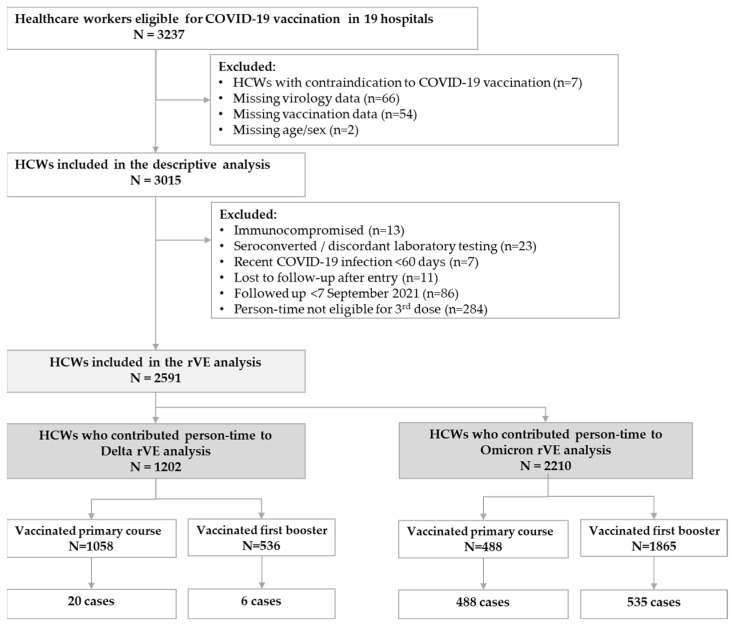
Data inclusion and exclusion flow chart, VEBIS HCW study, 2 May 2023.

After applying the exclusion criteria (Figure 1), we included in the cohort profile analysis 3015 HCWs (range per hospital 57–326), with a total follow-up time of 482,335 person-days, a median time of follow-up of 238 days (interquartile range (IQR) of 107–386), and a maximum follow-up time of 717 days. These HCWs were subsequently included in the incidence and vaccine effectiveness analyses (Figure 1).

Participating HCWs during the three waves were mostly female (2396, 79%), with a median age of 44 years (IQR 35–53) years; 559 (33%) presented with at least one underlying condition (Table 1). The most frequent of these conditions were hypertension (157, 9.2%), asthma (87, 5.1%), and rheumatic diseases (42, 2.5%); 933/2666 (35%) had a body mass index ≥ 25. A total of 1041/2933 (35%) HCWs reported at least one SARS-CoV-2 infection before enrolment in the study. Similar characteristics were reported by predominant waves, except for the higher proportion of HCWs with prior SARS-CoV-2 infection amongst those participating during the Omicron wave (Table 1).

**Figure 2:**
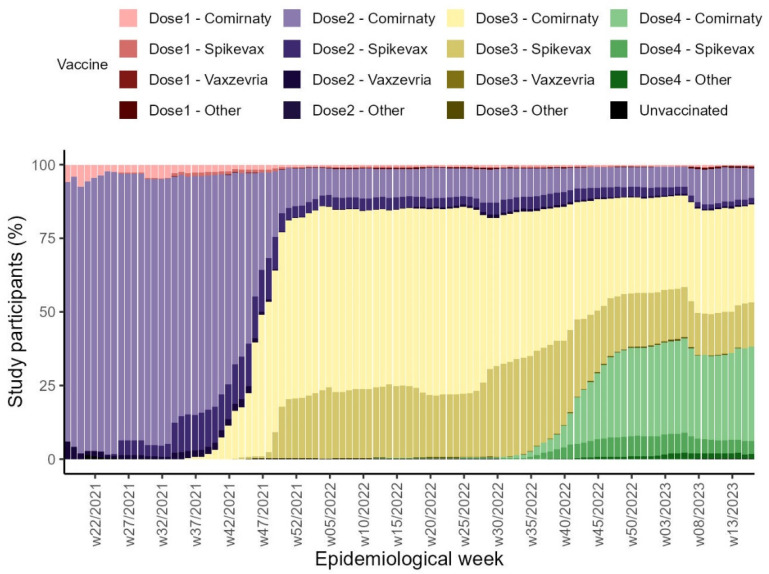
COVID-19 vaccine brands by dose and time, multi-country VEBIS HCW COVID-19 VE study, May 2021–May 2023.

In the pre-Delta period, 1337 HCWs participated from seven hospitals, and 1238 (93%) had already completed the primary course vaccination. Of the 1641 HCWs participating in the Delta wave, 1517 (92%) were vaccinated with primary course vaccination at enrolment and 896 (55%) HCWs received the first booster between September 2021 and December 2021. During the Omicron-dominated period, 1828 (72%) HCWs received the first booster; 110 (4%) HCWs had received a second booster dose at enrolment, and 413 (16%) were vaccinated with a second booster by the end of the follow-up. The most commonly used vaccine was mRNA BNT162b2 (Comirnaty, Pfizer/BioNTech) original monovalent vaccine, followed by mRNA-1273 (Spikevax, Moderna) original monovalent vaccine (Figure 2).

During the study period, 1050 SARS-CoV-2 infections were detected in 955 (31.7%) HCWs; 721 (69%) were symptomatic. Four HCWs were hospitalised for the COVID-19 episode during the follow-up, of whom two needed oxygen therapy. The four HCWs were vaccinated with two doses of Vaxzveria (one HCW), three doses of Spikevax monovalent original (one HCW), four doses of Comirnaty monovalent original (one HCW), and two doses of Vaxzveria vaccine, boosted with Comirnaty monovalent original vaccine (one HCW). Three of the four HCWs reported suffering from underlying conditions: one with cardiovascular disease, one with rheumatic disease, and one with diabetes and asthma.

### 3.2. Incidence of SARS-CoV-2 Infection by Wave

**Figure 3:**
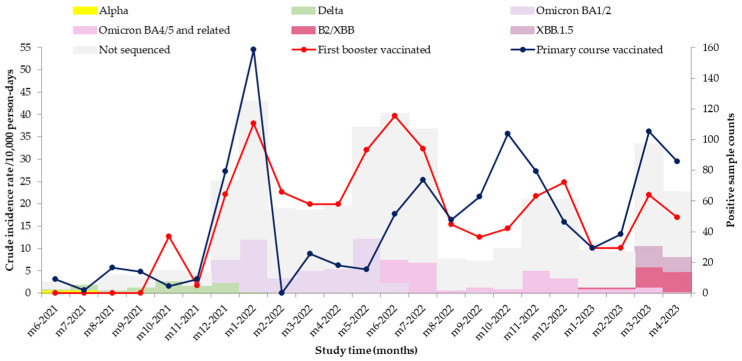
Incidence rate of laboratory-confirmed SARS-CoV-2 infection and number of samples sequenced in 11/19 sites, VEBIS HCW study, May 2021–May 2023.

During the pre-Delta period, we detected 14 SARS-CoV-2 infections with an incidence of 3.01/10,000 person-days (range/country: 1.18–4.97). During the predominant circulation of the Delta variant, we detected 32 SARS-CoV-2 infections, with an incidence of 4.21/10,000 person-days (range/country: 3.04–13.80). During the Omicron variant predominant circulation period, the incidence was 23.20/10,000 person-days overall (range/country: 8.46–41.79). The incidence was lower in the HCWs vaccinated with the first booster dose than in those vaccinated with primary course during autumn 2021 when the Delta variant circulated and autumn 2022 when Omicron BA4/5 circulated (Figure 3).

### 3.3. Relative Vaccine Effectiveness of the First Booster COVID-19 Vaccine Dose

During the predominant circulation of the Delta variant (7 September–14 December 2021), we included 1195 HCWs vaccinated with primary course, and 534 (45%) with the first booster dose (Figure 1 and Supplementary Table S1). rVE against SARS-CoV-2 infection was 59% (95% CI: −25; 86) and 62% (95% CI: −110; 93) against symptomatic infections (Table 2). No events were detected in HCWs followed up more than 3 months after the first booster within the Delta-predominant period.

During the predominant circulation of the Omicron variant (15 December 2021–2 May 2023), we included 496 HCWs vaccinated with primary course, and 1868 with the first booster dose (Figure 1 and Supplementary Table S1). rVE against infection was 22% (95% CI: 1; 39) and 29% (5; 47) against symptomatic infections. By time since the first booster dose, rVE against infection was 51% (95% CI: 30; 65) 7–89 days after the booster dose and 7% (95%CI: −22; 28) ≥90 days after the first booster dose. rVE against symptomatic infections was 55% (32; 70) 7–89 days after the first booster dose and 14% (−19; 37) ≥90 days after the first booster dose (Table 2).

## 4. Discussion

We present the results of a prospective cohort study in 19 hospitals from 10 European countries aimed at measuring SARS-CoV-2 infection incidence and COVID-19 VE in HCWs. Despite the high vaccine coverage for the primary course and first booster dose in our study, the participants were affected by the Omicron wave with a 5.5-fold higher SARS-CoV-2 infection incidence than in Delta and 7.7-fold higher than in pre-Delta periods. The rVE results suggest that HCWs who received a first COVID-19 vaccine booster dose were additionally protected against laboratory-confirmed infection compared to primary course vaccination alone. In the first 3 months following the first booster vaccination, we observed similar rVE point estimates during the predominant circulation of Omicron waves as in Delta, despite the wide confidence intervals due to the low number of events in the later period. Overall rVE against infection during the Omicron-predominant circulation period was generally lower, although still present, and was largely due to the greater decrease observed after 3 months from the first booster dose. This could be explained by both the waning protection with time since vaccination, following a decrease in protective antibodies and the antigenic difference between the vaccine and circulating strains. Indeed, the vaccine predominantly used in our study was based on the SARS-CoV-2 original strain offered intramuscularly, while the Omicron variant sub-lineages were found to escape vaccination-induced immunity.

Early evidence after the first booster introduction suggested that despite lower VE against Omicron variant sub-lineages [16,17,18], booster doses still offered additional benefits [19]. COVID-19 vaccine protection was reported to be higher against severe disease requiring hospitalisation [16,20]. The lower COVID-19 vaccine effectiveness was reported for the first booster dose against mild and asymptomatic infections [21,22] compared to symptomatic infections. In our study, only four HCWs needed hospitalisation during their COVID-19 episode, and the rVE point estimates for symptomatic HCWs were higher than those for asymptomatic HCWs in both Delta and Omicron periods.

The observed rVE point estimate against asymptomatic infection, though lower than rVE against symptomatic infection, is in line with other studies which indicate that well-timed vaccination programmes may help reduce SARS-CoV-2 transmission by decreasing infectiousness [23], albeit to a lower extent for Omicron variants compared to previously circulating variants [24]. Further studies on VE against transmission and the correlation of number of vaccine doses and time since last dose with viral load using the monovalent XBB vaccine and in the setting of currently circulating variants added important knowledge after this study. In HCWs, reducing transmission is crucial for protecting vulnerable patients. This is particularly important in the post-pandemic phase when only passive screening is recommended for respiratory infections among symptomatic HCWs, with subsequent self-reporting and isolation in order to avoid nosocomial transmission [25]. Besides vaccination, other protective measures such as regular testing and wearing protective equipment remain necessary in all HCWs, especially during the emergence of the new SARS-CoV-2 variant/sub-lineage strains, and regardless of the severity of the disease that these strains may cause.

We report lower rVE point estimates than those presented by others for the Delta- [19,21,26] and Omicron-dominant periods [19,21,26,27,28,29], mainly related to the different timing of the study and longer follow-up. However, our VEBIS HCW VE study has different methodological strengths, related to the multi-country study including 19 hospitals from 10 countries across Europe, with good geographical representation. We also used regular testing (with the exclusion of untested person-time and person-time with discordant serological and virology tests), and had comprehensive data collection (vaccination, prior SARS-CoV-2 infections, and other in-hospital and community exposures). In addition, we could adjust for variables known to be strong confounders and account for the susceptibility to infection by excluding the person-time after prior infection.

Our study also has several limitations. First, the number of participating HCWs was lower than recommended in some hospitals, and therefore we were unable to estimate the heterogeneity between the study sites. However, hospitals were included as fixed effects in all analyses to attempt to account for this variability. In addition, the low number of events resulted in imprecise estimates as reflected in the wide confidence intervals, especially during the Delta-predominant circulation period. Second, the participation of HCWs increased over time and acceptability for participation may have been related to the ability of participating laboratories to test saliva samples for SARS-CoV-2 by PCR. Saliva testing has been shown to perform well in comparison to nasopharyngeal swabs, particularly in the early stages of infection [30]. Third, the study was not powered to adjust for other possible confounders, although we collected additional information on different risk and protective factors that we can use to further understand the risk patterns in each hospital. Finally, our results are based on longer follow-up in some hospitals with different sub-lineages circulating in the Omicron period; this resulted in potential heterogeneity that we tried to mitigate by adjusting for hospital and calendar time in the regression analysis.

## 5. Conclusions

The vaccination of HCWs is recommended due to their high risk of occupational exposure, their work with vulnerable populations, and the necessity of maintaining services during periods of peak community transmission of respiratory infections. Our study adds to the body of evidence that the emergence of the Omicron variant led to a higher incidence of mild infection among HCWs and that the vaccination with the first booster COVID-19 vaccine dose offered additional protection against SARS-CoV-2 infection compared to primary course vaccination for about three months and started to decrease after that. In addition, our findings indicated that vaccination needs to be implemented timely along with other preventive measures prior to any anticipated wave of infection to offer the best protection to HCWs and subsequently to their patients. However, additional research is needed to investigate potential improvements of COVID-19 vaccines and to identify the optimal timing for (re)vaccination.

The VEBIS HCW cohort could be a powerful platform to further monitor and investigate the effectiveness of vaccines recommended to HCWs, and to inform key public health interventions for this high-risk population.

## Figures and Tables

**Table 1:** Participant HCW description at enrolment or the start of the study period, VEBIS HCW study, 2 May 2023.

Characteristics	Overall		Pre-Delta		Delta		Omicron	
	(*n* = 3015)		(*n* = 1337)		(*n* = 1641)		(*n* = 2554)	
**Sex**	N	%	N	%	N	%	N	%
Female	2396	79	991	74	1230	75	2096	82
**Age (years**)								
Median [p25–75]	44	35–53	45	35–54	45	35–54	44	35–53
<35	741	25	329	25	384	23	598	23
35–40	383	13	163	12	207	13	334	13
40–44	398	13	158	12	198	12	356	14
45–49	452	15	179	13	225	14	395	15
50–54	418	14	182	14	243	15	357	14
55+	623	21	326	24	384	23	514	20
**Role**								
Medical doctor	686	23	345	26	428	26	536	21
Nurse	1361	46	597	45	751	46	1184	47
Allied professionals	119	4	50	4	59	4	90	4
Laboratory	149	5	81	6	85	5	132	5
Administration/reception	324	11	121	9	138	8	285	11
Ancillary	75	3	48	4	48	3	55	2
Other	249	8	86	6	121	7	225	9
**Smoking**								
Never smoked	1632	55	698	53	890	55	1411	56
Ex-smoker	635	21	302	23	351	22	552	22
Current smoker	689	23	309	24	364	23	552	22
Missing	59	2	28	2	36	2	39	2
**Underlying conditions**								
At least one	559	33	214	36	265	37	525	35
**Previous SARS-CoV-2 infection**								
Yes	1041	35	169	13	244	15	1002	40
No	1892	65	1151	87	1376	85	1482	60
Missing	82	3	17	1	21	1	70	3
**Vaccinated (doses)**								
0	84	3	37	3	38	2	45	2
1	96	3	62	5	58	4	47	2
2	1818	60	1238	93	1517	92	524	21
3	907	30			28	2	1828	72
4	110	4					110	4

N = number of HCWs with the respective characteristic; p = percentile.

**Table 2:** Adjusted relative vaccine effectiveness of the first booster COVID-19 vaccination by period of predominant circulation of Delta and Omicron variants and outcome, multi-country VEBIS HCW study, 2 May 2023.

Outcome	Delta-Predominant Circulation					Omicron-Predominant Circulation				
				Adjusted by Site	Fully Adjusted *				Adjusted by Site	Fully Adjusted *
	N	PT	e	rVE (95% CI)	rVE (95% CI)	N	PT	e	rVE (95% CI)	rVE (95% CI)
**All infections**										
***Overall***										
Primary course ≥ 90 days	1058	36,990	20	reference	reference	488	35,916	88	reference	reference
First booster dose ≥ 7 days	536	13,046	6	53 (−43; 85)	59 (−26; 87)	1864	197,822	535	7 (−18; 27)	22 (1; 39)
***By time since vaccination***										
Primary course ≥ 90 days	1058	36,990	20	reference	reference	488	35,916	88	reference	reference
First booster 7–89 days	536	13,016	6	53 (−44; 85)	59 (−26; 87)	1008	48,969	135	43 (21; 60)	51 (31; 65)
First booster ≥90 days	7	30	0	N/A	N/A	1537	148,853	400	−13 (−46; 23)	6 (−23; 28)
**By infection type**										
***Asymptomatic HCWs***										
Primary course ≥ 90 days	1058	36,990	7	reference	reference	488	35,916	27	reference	reference
First booster dose ≥ 7 days	536	13,046	4	46 (−146; 88)	59 (−88; 91)	1864	197,822	197	1 (−52; 35)	10 (−38; 41)
***By time since vaccination asymptomatic HCWs***										
Primary course ≥ 90 days	1058	36,990	7	reference	reference	488	35,916	27	reference	reference
First booster 7–89 days	536	13,016	4	45 (−146; 88)	59 (−88; 91)	1008	48,969	45	37 (−15; 66)	43 (−6; 69)
First booster ≥ 90 days	7	30	0	N/A	N/A	1537	148,853	152	−20 (−93; 25)	−9 (−76; 33)
***Symptomatic HCWs***										
Primary course ≥ 90 days	1058	36,990	13	reference	reference	488	35,916	61	reference	reference
First booster dose ≥ 7 days	536	13,046	2	60 (−117; 93)	62 (−114; 93)	1864	197,822	338	10 (−19; 33)	29 (5; 47)
***By time since vaccination symptomatic HCWs***										
Primary course ≥ 90 days	1058	36,990	13	reference	reference	488	35,916	61	reference	reference
First booster 7–89 days	536	13,016	2	60 (−117; 93)	62 (−114; 93)	1008	48,969	90	47 (20; 64)	55 (33; 70)
First booster ≥ 90 days	7	30	0	N/A	N/A	1537	148,853	248	−9 (−49; 20)	14 (−19; 37)
**By prior SARS-CoV-2 infection**										
***Prior infection***										
Primary course ≥ 90 days	168	6225	1	reference	reference	267	25,203	47	reference	reference
First booster dose ≥ 7 days	49	1320	0	N/A	N/A	744	81,887	146	27 (−3; 48)	20 (−14; 43) **
***No prior infection***										
Primary course ≥ 90 days	885	30,594	19	reference	reference	221	10,355	41	reference	reference
First booster dose ≥ 7 days	484	11,687	6	58 (−31; 87)	57 (−36; 86) **	1182	114,005	378	19 (−15; 43)	28 (−2; 49) **

* Adjusted by age, sex, site, at least one underlying condition, and prior SARS-CoV-2 infection; ** Adjusted by age, sex, site, and at least one underlying condition. N: number of HCWs; PT: person-days; e: events; rVE: relative vaccine effectiveness; N/A: not applicable.

## Data Availability

Data will be made available on request.

## Supplementary Materials

The following supporting information can be downloaded at: https://www.mdpi.com/article/10.3390/vaccines12111295/s1. Table S1. Description of participant healthcare workers (eligible for the first booster dose) by predominant circulation periods of Delta and Omicron variants, multi-country VEBIS HCW VE study, September 2021–May 2023.
