# Supplementary material for: Incidence of SARS-CoV-2 Infection Among European Healthcare Workers and Effectiveness of the First Booster COVID-19 Vaccine, VEBIS HCW Observational Cohort Study, May 2021–May 2023
Source: Vaccines (Basel). 2024 Nov 19;12(11):1295. doi: 10.3390/vaccines12111295 (PMC11598658; doi:10.3390/vaccines12111295)
Supplement: Supplementary file 1 [file vaccines-12-01295-s001.zip › vaccines-3263125-supplementary.pdf]

vaccines

MDPI

Supplementary Table S1: Description of participant healthcare workers (eligible for the first booster dose) by predominant circulation periods of Delta and Omicron variants, multi-country VEBIS HCW VE study, September 2021-May 2023.

	Delta-predominant circulation period				Omicron-predominant circulation period			
	Eligible for the first booster dose		Received the first booster dose		Eligible for the first booster dose		Received the first booster dose	
	TOTAL (n=1202)	%	TOTAL (n=536)	%	TOTAL (n=2210)	%	TOTAL (n=1864)	%
**Gender**								
Woman	902	75	394	74	1818	82	1516	81
**Age (years)**								
Median [p25-75]	45	35–53	46	36–54	44	35–53	44	35–53
>35	289	24	107	20	519	23	440	24
35-40	159	13	72	13	292	13	246	13
40-44	148	12	76	14	302	14	252	14
45-49	163	14	70	13	356	16	301	16
50-54	189	16	92	17	306	14	246	13
55+	254	21	119	22	435	20	379	20
**Role**								
Medical Doctor	332	28	172	32	450	21	409	22
Nurse	498	42	212	40	1055	48	859	47
Allied professionals	53	4	19	4	78	4	62	3
Laboratory	76	6	30	6	115	5	103	6
Administration/Reception	106	9	42	8	237	11	198	11
Ancillary	38	3	15	3	48	2	40	2
Other	95	8	43	8	196	9	165	9
**Underlying conditions**								
At least one	178	35	92	42	463	36	393	37
**Previous SARS-CoV-2 infection**								
Yes	173	14	49	9	865	40	653	36
No	1021	86	484	91	1311	60	1182	64
Missing	8	1	3	1	34	2	29	2
**Brand of the booster dose**								
Comirnaty - Monovalent (Pfizer/Biotech)			487	91			1,434	77
Spikevax - Monovalent (Moderna)			46	9			413	22
Other			3	<1			17	1
